# Exercise in an electrotactic flow chamber ameliorates age-related degeneration in *Caenorhabditis elegans*

**DOI:** 10.1038/srep28064

**Published:** 2016-06-16

**Authors:** Han-Sheng Chuang, Wan-Jung Kuo, Chia-Lin Lee, I-Hua Chu, Chang-Shi Chen

**Affiliations:** 1 Department of Biomedical Engineering, National Cheng Kung University, Taiwan; 2 Medical Device Innovation Center, National Cheng Kung University, Taiwan; 3 Department of Sports Medicine, Kaohsiung Medical University, Taiwan; 4 Department of Biochemical and Molecular Biology, National Cheng Kung University, Taiwan

## Abstract

Degeneration is a senescence process that occurs in all living organisms. Although tremendous efforts have been exerted to alleviate this degenerative tendency, minimal progress has been achieved to date. The nematode, *Caenorhabditis elegans* (*C. elegans*), which shares over 60% genetic similarities with humans, is a model animal that is commonly used in studies on genetics, neuroscience, and molecular gerontology. However, studying the effect of exercise on *C. elegans* is difficult because of its small size unlike larger animals. To this end, we fabricated a flow chamber, called “worm treadmill,” to drive worms to exercise through swimming. In the device, the worms were oriented by electrotaxis on demand. After the exercise treatment, the lifespan, lipofuscin, reproductive capacity, and locomotive power of the worms were analyzed. The wild-type and the Alzheimer’s disease model strains were utilized in the assessment. Although degeneration remained irreversible, both exercise-treated strains indicated an improved tendency compared with their control counterparts. Furthermore, low oxidative stress and lipofuscin accumulation were also observed among the exercise-treated worms. We conjecture that escalated antioxidant enzymes imparted the worms with an extra capacity to scavenge excessive oxidative stress from their bodies, which alleviated the adverse effects of degeneration. Our study highlights the significance of exercise in degeneration from the perspective of the simple life form, *C. elegans*.

With the advancements in modern medicine, the growing senior population has progressively reshaped global demographics. This tendency raises urgent demand to deal with the increase of degenerative diseases, which can ensure a better quality of senescent life among this population. Although advanced surgical and pharmaceutical technologies have rapidly developed in recent years, degeneration remains an irreversible process. Given that early prevention is always better than cure, exercise is considered an effective and simple measure in preventing degenerative diseases. A recent study reported that exercise can regulate brain-derived neurotrophic factor (BDNF) and serotonin (5-HT) to treat Alzheimer’s disease (AD)^1^. Another model that addresses the relationship of oxidative stress and antioxidants has proven that the antioxidant defense system can be enhanced through regular exercise (Supplementary Fig. 1 and Note 1)^2^. However, the rise of reactive oxygen species (ROS) has resulted in skepticism among researchers with regards to the benefits of exercise^3^. Consequently, the complicated nature of exercise in improving age-related degeneration remains controversial.

*Caenorhabditis elegans* (*C. elegans*) is a simple model animal that shares over 60% genetic similarities with humans. Therefore, studying the effects of exercise on *C. elegans* can provide valuable information on improving the physical health and psychological well-being of society, specifically the elder population. Age spots, such as lipofuscin (LF), can indicate ROS production, and is typically regarded as a complex substance containing damaged mitochondrial remains, misfolded/unfolded proteins, waste lipids, and few metals resulting from oxidative stress^4^,^5^. Abnormal LF accumulation is also frequently found in morbid tissues, such as the brains of AD patients^6^. Similarly, high LF concentration indicates cellular impairment in *C. elegans* associated with high occurrences of degenerative disorders^7^,^8^. However, prior evidence^9^ have shown that exercise can reverse or alleviate this process by increasing the cellular capacity that is required to deal with oxidative stress. According to the finding, we expect to observe rejuvenescent phenotypes, such as decreased LF accumulation and longevity, on the exercise-treated worms. To investigate the degenerative degree of *C. elegans* with and without exercise, several physiological (lifespan, brood size, and appearance) and biomechanical (swimming gait and locomotive power) properties were analyzed.

Electrotaxis was used herein as a driving force to keep worms constantly swimming in the flow chamber within a desired timeframe. The Bristol wild-type (N2) and the transgenic strains (CL2120, Aβ accumulation) were bred on agar dishes at 20 °C and 15 °C, respectively, prior to treatment and were transferred to a chamber filled with nematode growth media (NGM) during the treatment. The worms were forced to exercise for 10 min daily starting from L3 to adult day 6. According to the stress response of the transgenic strain, TJ356 [*daf-16p::daf-16a/b::GFP*+ *rol-6*], the electrotaxis-induced stress on the worms was only significant during the initial stage; it became the same as the control group afterward. A comparison of worm motility on the agar dish and the worm treadmill showed that worms became vibrant when staying in the flow chamber. The swimming locomotion is therefore defined as an “exercise state”. Followed by the exercise treatment, the locomotive power, lifespan, and progeny size of both CL2120 and N2 strains all increased. In addition, exercise-treated worms exhibited dense mitochondria and rapid muscle recovery. Results imply that the exercise-treated worms are likely to live with younger and healthier states compared with their untreated counterparts. This study successfully presented an electrotactic flow device that can drive *C. elegans* to swim on demand. The treatment effectively alleviated the degenerative behaviors and phenotypes in the worms. Accordingly, LF accumulation and ROS level were reduced. This reduction and the accompanying physiological improvements support the exercise-antioxidant relationship and negate the likelihood of adverse effects resulting from exercise.

## Results

### The worm treadmill system: experimental setup and operation

**Figure 1:**
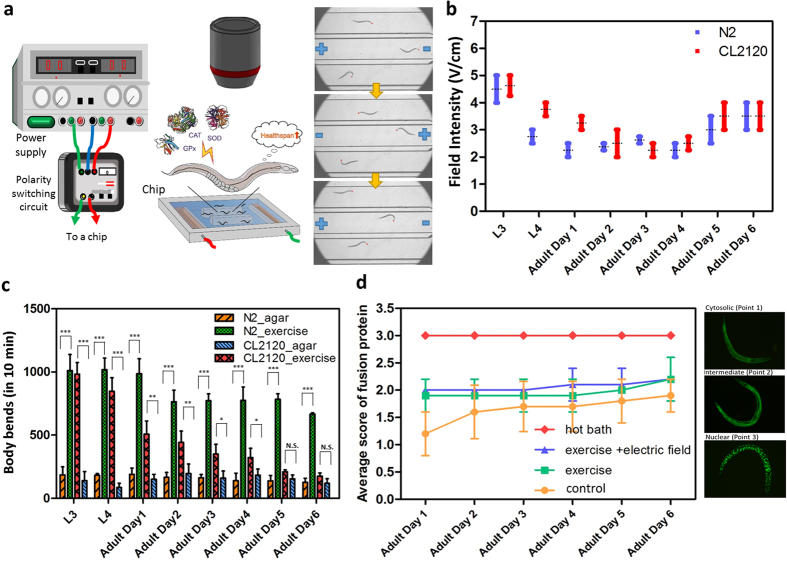
Worm treadmill system for conducting the exercise treatment on live *C. elegans*. (**a**) Worm treadmill system consisting of a flow chamber, a microscope, a DC power supply, and a polarity switching circuit. The locomotion of the worm is driven by mild electrotaxis in the microchannels. Antioxidant enzymes, such as SOD, CAT, and GPx, are induced by the surge of oxidative stress through exercise. The consecutive images on the rightmost column show that worms are attracted to the negative pole in a DC electric field. The red dots are the heads of the worms. (**b**) Variations of effective electric intensities with respect to different developmental stages of *C. elegans*. The electric intensity declines from the larval to adult stages because of neuronal maturity. The trend is reversed after adult day 4 owing to neuronal degeneration. (**c**) Comparison of the body bends of worms in the flow chamber and on the agar. The high body bends are a clear sign of vigorous exercise. However, the exercise behavior of CL2120 weakens after adult day 6. The weakened swimming gait is affected by the progressive accumulation of beta amyloid peptides (Aβ). (**d**) A plot of the stress responses of hot bath, pure exercise, exercise with electrotaxis, and control (bred on agar) groups. A total of 320 transgenic TJ356 worms were identified under a fluorescent microscope. The phenotypes were classified as “cytosolic” (score 1), “intermediate” (score 2), and “nuclear” (score 3) according to their nuclear localization.

The flow chamber, called “worm treadmill,” consists of acrylic microchannels, electrical wires, and a cover glass (Fig. 1a and Supplementary Fig. 2). Microchannels were fabricated by using a CNC mill (EGX-400, Roland) and each channel measured 1 cm in width, 1.5 mm in depth, and 2 cm in length. Electrodes were installed 5 cm apart at both ends of the microchannels. A direct current (DC) power source (QWINSTEK, GPC-3030DQ) was connected to the electrodes to ensure that the worms were constantly swimming in the microchannels for the prescribed duration. A home-made polarity switching circuit was used to periodically change the electrical polarity. Given that *C. elegans* is attracted toward the cathode by electrotaxis, switching the polarity of the electric field can confine the worm’s activity within a finite space. The exercise (i.e., swimming) duration was experimentally set to 10 min because a high fraction of worms (both N2 and CL2120) were injured when the duration exceeded 10 min. Agarose gel was used to cover the wires so as to avoid air bubble formation. The electric field was maintained below 5 V/cm.

Microchannels were initially filled with a diluted NGM buffer (NGM:DI water = 1:3) during the electrotactic operation and then loaded with worms. The top of the flow chamber was covered with a glass lid to confine the worms as well as the electric field. The entire flow chamber was subsequently placed under a microscope (IX71, Olympus) to monitor the worms swimming for 10 min. Electrical polarity was switched every 30 to 60 s. Exercise treatment, herein, refers to the 10 min uninterrupted swimming of worms guided by the electrotaxis. The exercise treatment was performed to the same batches of worms once per day for a total of 8 days from the L3 stage to adult day 6. Notably, the Alzheimer model strain, CL2120, showed a high rate of paralysis after adult day 6. Worms were transferred to another fresh 60-mm agar dishes with *E. coli* OP50 after each exercise treatment and were cultured in a temperature-regulated incubator. Several physiological (LF accumulation, progeny, lifespan, and body bends), biochemical (reactive oxygen species, sarcomere phenotype, and mitochondria), and biomechanical (unit kinetic power) properties were recorded after every treatment until the end of the experiment.

### Movement and electrotaxis of *C. elegans* in the flow chamber

Prior studies^10^,^11^ have stated that *C. elegans* can respond to electrical stimulation on open agar gel surfaces and move toward the negative pole. This behavior, known as electrotaxis, is highly reproducible and sensitive to the direction and strength of the electrical signal. Although the biological relevance of the worm’s response to electrotaxis remains unclear, it has been suggested that this behavior may evolve into a host finding cue in parasitic nematodes^10^. Taking advantage of the electrotaxis of *C. elegans*, a low-voltage electric field can be used as an attractive source to drive the worms to swim on demand (Supplementary Fig. 3 and Supplementary Video 1). However, electrotaxis is not always effective on *C. elegans* at all stages. We observed that young larval worms (L1 and L2) may display an obscure response to external electric fields because of their immature electrosensory system^12^. The minimum electrical intensity of electrotaxis at different developmental stages was investigated to avoid unnecessary neuronal damage from over electrical stimulation (Fig. 1b). The minimum electrical intensity requirements for N2 and CL2120 were nearly the same. The minimum electrical intensity declined from 4 V/cm to as low as 2 V/cm at the L3 stage to adult day 4, but slightly increased to 3 V/cm at older adult stages. The reversed electrical response can be attributed to a mature nervous system at the young adult stages, which then became degenerative and impaired during the old adult stages. When the worms were exposed to electric fields at voltages higher than the maximum thresholds, most worms exhibited paralytic behaviors, such as the curling or trembling of their bodies. The response time of *C. elegans* to electrical signal was swift and robust. Therefore, changing the electrical polarity resulted in an immediate reversal of a worm’s direction of movement. This characteristic allows us to confine worms within the microchannels by simply switching the electrical polarity alternately.

Considering the differences of *C. elegans* from higher animals in terms of appearance, their exercise states were redefined in the current study based on their locomotory gait. Worm motility can be observed by counting the cycle of a worm’s head thrashing trajectory, which is called “body bends.” Prior studies^13^,^14^,^15^,^16^ have shown that *C. elegans* performs swimming and crawling gaits at low and high medium viscosities, respectively. Therefore, we can expect worms to show vigorous locomotion in the flow chamber. Although *C. elegans* appears to swim in an aquatic environment without an electric field, its behavior is unpredictable and uncontrollable. Ghosh *et al*.^17^ stated that *C. elegans* exhibits episodic swimming in a liquid medium. Belfer *et al*.^18^ also reported the quiescent behavior of *C. elegans* confined in aqueous droplets. To avoid the abovementioned uncertainties, electrotaxis was used in the current study to ensure that worms would constantly swim within a desired timeframe. The high motility of *C. elegans* in the flow chamber guided by electrotaxis is regarded as the unique “exercise state” for *C. elegans* in this research. In contrast, mild crawling on an agar dish is considered as the “normal state.” The body bends of N2 and CL2120 worms in 10 min were separately measured in the NGM medium and on agar dishes (Fig. 1c). Results showed that the exercise group had higher body bends than the normal group across all developmental stages, suggesting that the flow chamber can serve as a “worm treadmill” for *C. elegans.* However, the body bends degraded for both strains as the stages developed. Especially for CL2120, the body bends of the exercise group approached that of the normal group after adult day 6. Accordingly, the exercise treatment was halted after adult day 6.

The treated worms were physiologically examined after each exercise treatment from L3 to adult day 6. Neither physiological nor physical damages were observed. The treated worms constantly resumed their normal behaviors, such as forage, reproduction, and sinusoidal movement, when placed on agar dishes with *E. coli*. In addition, fecundity and lifespan, along with a number of basic sensory neuronal systems (e.g., light sensitive, mechanosensory, and chemosensory neurons), were confirmed as normal. Light sensitivity, mechanosensory, and chemosensory responses were checked by blue light, physical touch, and food attraction. Nevertheless, TJ356, a transgenic strain carrying DAF-16::GFP, was used to monitor the stress perception inside the worm’s body. Under a stressful condition, the FOXO transcription factor DAF-16 will cause nuclear localization resulting in granular green fluorescence. To score the stress expressions, the fluorescent patterns were classified into three categories, namely, cytosolic, intermediate, and nuclear, by considering the major localization of the DAF-16::GFP fusion protein (Fig. 1d). Measurement started from adult days 1 to 6. A hot bath was used to induce full nucleation by heating worms at 33 °C water for 30 min. Pure exercise is defined as free swimming without electrotaxis. The flow chamber was tapped by hands periodically to prevent worms from pausing or fully stopping. A control group was measured on an agar dish. Throughout the measurement, the pure exercise and exercise groups with electrotaxis exhibited mild stress, whereas those in the hot bath group indicated the strongest stress at all times. In contrast, worms in the control group began with nearly no stress and ended up with mild stress at adult day 6. This tendency implies that the aging process imposes stress on worms as well. Compared with the control and pure exercise groups, the stress resulting from the exercise with electrotaxis seemed to be negligible after adult day 2, suggesting that exercise and electrotaxis should cause no harm to the worms.

### Effects of exercise on sarcomeres and mitochondria formation in aging *C. elegans*

Unlike humans, the degeneration phenotypes on *C. elegans* are quite different. Progressive locomotory impairment during *C. elegans* aging is one of the visible indicators. The Alzheimer model strain, CL2120, causes paralysis by expressing their Aβ in muscles. The degeneration and morbidity then lead to the loss of muscular functions. In search of an evidence for such age-related muscle deprivation, the transgenic strain, RW1596 [*myo-3p::GFP::myo-3*], with GFP-tagged MYO-3 proteins that can localize in the body wall sarcomeres was employed. Muscle degeneration was evaluated through the patterns and smoothness of muscle fibers. Five categories, scored from 0 to 4, were used to define the status of the body wall sarcomere integrity (Supplementary Fig. 4a). Point 0 shows that the sarcomeres are smooth, tightly arranged, and in parallel position. On the contrary, point 4 indicates that the sarcomeres are ruptured, fragmental, and randomly distributed. A final score was obtained from the average of the total worms evaluated. Scores were recorded daily from adult days 2 to 15 (Supplementary Fig. 4b). Micrographs were immediately captured after every exercise treatment.

**Figure 2:**
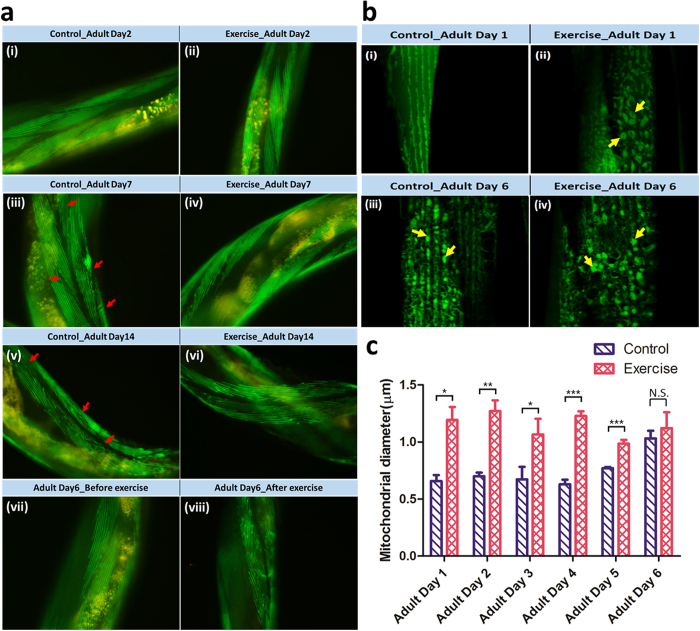
Investigation of the body wall sarcomeres and mitochondria in *C. elegans* before and after exercise. (**a**) Micrographs of body wall sarcomeres at adult days 2, 7, and 14. Sub-images (**i**–**vi**) were acquired with a 100× oil immersion objective. The transgenic strain, RW1596 [*myo-3p*::GFP::*myo-3*], was used. Sub-images (**vii**) and (**viii**) were acquired before and after exercise, respectively. Some of the muscle fibers are indicated by the red arrows. (**b**) Micrographs of mitochondria changes between the untreated and exercise-treated *C. elegans*. A transgenic strain, SJ4103[*myo-3p*::GFP(mit)], was employed. The sub-images (**i**,**ii**) and (**iii**,**iv**) show that the mitochondrial morphology becomes larger and denser after exercise at adult day 1, however, it compromises at adult day 6. A number of the fused mitochondria are indicated by the yellow arrows. (**c**) Plot of the mean diameters of mitochondria varying from adult days 1 to 6 (n = 40). The mean diameter of the mitochondrion is larger during the early adult stages in the exercise-treated than the untreated worms. However, this improvement is compromised as the sarcomere integrity downgrades because of age-related degeneration.

Statistical results unexpectedly showed that the sarcomeres were more organized in the untreated than the exercise-treated worms at their young adult stages (Fig. 2a_i; Supplementary Fig. 4c,d). However, as the worms aged, the sarcomeres progressively became rather disorganized and loosely packed in the untreated worms than the exercise-treated worms (Fig. 2a_iii; Supplementary Fig. 4c,d). At adult day 14, the sarcomeres in both strains further degenerated with impaired functions and appearances compared with their young stages (Fig. 2a_v; Supplementary Fig. 4c,d). Despite the insignificance in their appearance, the exercise-treated worms seemed to maintain a better sarcomere condition than their untreated counterparts at later adult stages. Results indicated that, like humans, aging *C. elegans* also experienced the progressive loss of muscular mass and functions over time. However, the status of muscle fibers can be upregulated by appropriate exercise training. Notably, no wave-like muscle fibers (i.e., damage) were observed in the exercise-treated worms. Morphological phenotypes enabled the exercise-treated worms to exert increased energy for locomotion, forage, and reproduction. Compared with the muscle micrographs captured right after the exercise treatment at adult day 6, the appearance of sarcomeres was uneven and ruptured (Fig. 2a_vi). Surprisingly, the damaged sarcomeres rapidly recovered in just a few hours. More importantly, the recovered sarcomeres appeared to have become denser and stronger than the undamaged ones. Thus, the sarcomeres were endowed with heightened strength and power during the repeated damage-recovery process.

Generally, the vigorous locomotion of the exercise-treated worms typically appears with the presence of dense and fused mitochondria in their body wall muscle cells. The increased and enlarged mitochondria are regarded as a supportive evidence of healthy cells, which implies improved sarcomere functions. By employing the transgenic strain, SJ4103 [*myo-3p*::GFP(mit)], in the flow chamber, the morphology of mitochondria can be assessed with green fluorescence. A confocal microscope (FV1000, Olympus) equipped with a 300× objective was used to capture clear images of the mitochondrial granules because the typical size of a mitochondria is in sub-microns (Fig. 2b). To cope with the diverse mitochondrial morphology, the mean diameter of the mitochondrion was calculated using the hydraulic diameter (defined as a 4-fold area of each mitochondria image divided by its perimeter). Analysis was conducted on ImageJ (http://imagej.nih.gov/ij/). Mitochondrial morphology has been considerably discussed in prior literature^19^,^20^,^21^. Although mitochondria are highly dynamic organelles that constantly fuse and divide, their fusion can generally be observed in healthy cells. However, fission leads to unhealthy or apoptotic cells. In the current study, the mean diameters of mitochondria across all exercise-treated and untreated worms were assessed from L4 stage to adult day 6 (Fig. 2c). The results showed that exercise-treated worms certainly produced more and larger mitochondria than the untreated ones at the young adult stages. The trend is in good agreement with the observation in sarcomeres. Furthermore, mitochondrial morphologies at 0 h and 6 h after each exercise were compared. More and denser fused mitochondria were found after 6 h rather than 0 h, implying that a longer time was necessary to convert exercise benefits into sarcomeres (Supplementary Fig. 5a,b). When the worms were at the later developmental stages, the improvement appeared to be compromised. Such discontinuation can be attributed to muscle impairment resulting from age-related degeneration. Nevertheless, the early improvement proved to be sufficient in boosting all the body functions of worms, resulting in their extended lifespan, increased progeny, and lower oxidative stress.

### Kinetic power of exercise-treated *C. elegans*

**Figure 3:**
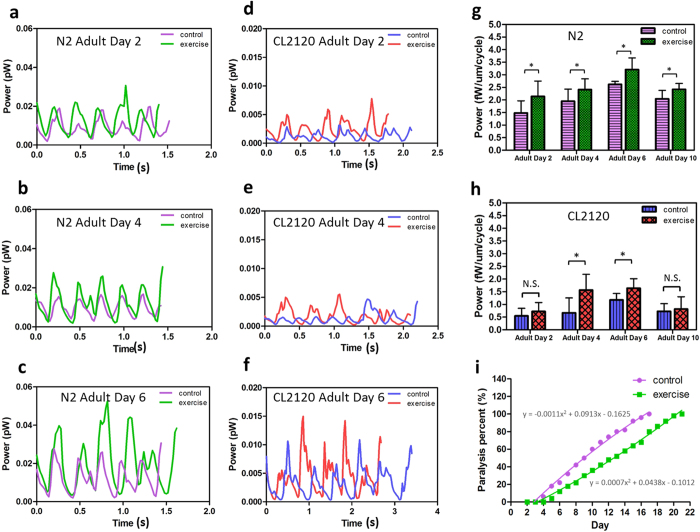
Plots of temporal kinetic power variations of N2 and CL2120 with and without the exercise treatment. (**a–c**) Kinetic power variations of N2 in three swim cycles measured at adult days 2, 4, and 6, respectively. The kinetic power of the exercise-treated worms is higher than that of the untreated worms. (**d–f**) Kinetic power variations of CL2120 in three swim cycles measured at adult days 2, 4, and 6, respectively. The kinetic power of the exercise-treated worms is higher than that of the untreated worms. However, the overall power remains inferior to the N2 worms. (**g**) Evolution of kinetic power per cycle for the exercise-treated and untreated N2 worms (n = 8). *denotes that p < 0.05 based on the Mann-Whitney U test. (**h**) Evolution of kinetic power per cycle for the exercise-treated and untreated CL2120 worms (n = 8). *denotes that p < 0.05 based on the Mann-Whitney U test. (**i**) Paralysis rate of the exercise-treated and untreated CL2120 worms (n = 50). Fifty worms were counted in each group (with or without exercise). Measurement stopped when all worms perished. The paralysis progress is evidently improved after the exercise treatment.

An image-based technique^22^ was used herein to noninvasively assess the kinetic power of *C. elegans* because many phenotypic changes are associated with the locomotory gait. This technique depends on the micro particle image velocimetry (μPIV)^23^,^24^. Specifically, the force exerted by the worm can be measured based on energy conservation by confining a worm in an aqueous droplet. To obtain the fluid motion, the droplet must be seeded with tracer particles (3 μm, Thermofisher Scientific), such that the flow field can be visualized with an epi-fluorescent microscope (Supplementary Fig. 6a). An acceleration field can be obtained when the flow velocities are delineated. Subsequently, the temporal kinetic power can be derived from the acceleration field over time (Supplementary Fig. 6b). Detailed information about the algorithm have been discussed in the prior literature^22^. The exercise-treated worms at adult days 2, 4, 6, and 10 were computed in order to understand the motility changes regulated by exercise (Fig. 3a–g). To avoid the size effect, power was divided by each worm’s body length. The kinetic power in a swim cycle exhibits a trend similar to the letter “W.” The fluctuation corresponds to the sinusoidal swimming gait of a worm. When the power reaches the bottom, the worm recoils its body to store energy, whereas it releases the energy for propulsion by stretching its body, resulting in the maximum power. In general, considering the progressive accumulation of Aβ in their sarcomeres, CL2120 worms exert lower power than N2 worms. Moreover, exercise-treated N2 and CL2120 worms all exhibited higher kinetic power than their control counterparts (Fig. 3h,i). Motility rejuvenation can last until adult day 6, but this can weaken afterwards.

Paralysis refers to locomotory morbidity relevant to the onset of degenerative diseases. The progress of paralysis over time was assessed by CL2120. Paralysis is defined in the current study as the silence of the worm’s tail (Supplementary Fig. 6c). A total of 50 worms were included in the control and exercise groups. The percentage of paralyzed worms over the total 50 worms was counted daily from adult day 2 until the death of all worms. The exercise group evidently exhibited a much slower onset of Alzheimer’s disease than the control group. Along with the improvement, the LF accumulation in CL2120 was effectively reduced. The improvement of paralysis is in good agreement with the previous kinetic power escalation. Thus, the result confirms that the onset of Alzheimer’s disease on CL2120 can be delayed with exercise.

### Physiological and biochemical phenotypes of exercise-treated *C. elegans*

**Figure 4:**
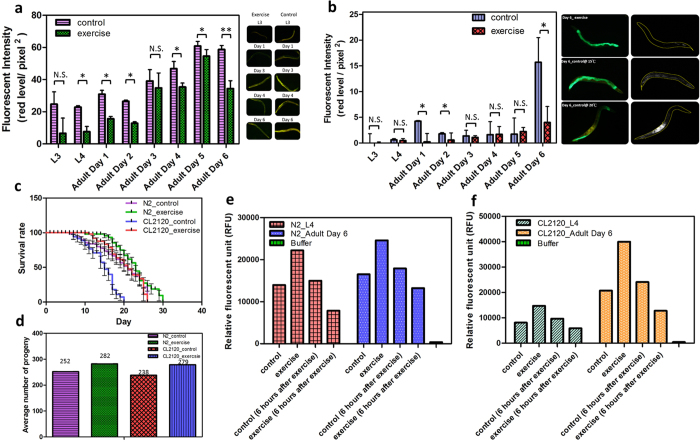
Physiological and biochemical phenotypes of *C. elegans* with and without the exercise treatment. (**a**) Plot of the fluorescent intensity of N2 stained with Nile red (n = 12). The LF levels are effectively mitigated at all developmental stages after exercise. The insets in the right column are corresponding images of the worms from the L3 stage to adult day 6. (**b**) Plot of the fluorescent intensity of CL2120 stained with Nile red (n = 8). Similar to the N2, the LF levels are mitigated at most developmental stages after exercise. A number of obscure results are disturbed by the green auto-fluorescence. The color insets in the right column are images illustrating the differences in LF accumulation among the worm bodies with and without the exercise treatment. The black and white insets are the color images as they are filtered off with a green palette. (**c**) Survivorship of the N2 (n = 30) and CL2120 (n = 30) worms. The lifespan based on a 50% survival rate for the exercise-treated and untreated N2 are 23 and 21, respectively. Worms were raised at 20 °C. The lifespan based on a 50% survival rate for the exercise-treated and untreated CL2120 are 19 and 15, respectively. Worms were raised at 15 °C. (**d**) The average progeny numbers of the N2 (n = 30) and CL2120 (n = 30) worms. Eggs were counted every day. The average progeny number was obtained from the total eggs divided by the number of worms. (**e,f**) Plots of the ROS levels of the N2 (n = 50) and CL2120 (n = 50) worms. For both strains, ROS surges right after exercise but drops rapidly after 6 h. A total of 50 worms were sonicated and stained with DCF-DA to measure their ROS levels on a microplate reader. CL2120 worms indicated higher ROS levels than their wild-type counterparts owing to the progressive accumulation of Aβ in the body.

Abnormal LF accumulation is frequently found in morbid tissues^7^,^8^,^25^. LF is typically regarded as a complex substance containing damaged mitochondrial remains, misfolded/unfolded proteins, waste lipids, and metals resulting from oxidative stress. Seriously cross-linked material renders LF immovable by any existing enzymes and intracellular scavenging processes, eventually leading to cell apoptosis. Numerous studies have reported the strong link between LF and morbid aging tissues^8^. According to the oxidative model, LF can either indicate or cause some degenerative diseases^7^. Therefore, the existence of LF granules is likely to indicate the severity of degeneration. To analyze the relationship between LF and the exercise treatment, the N2 and CL2120 worms were stained with the Nile red dye on a daily basis from L3 to adult day 6 in order to measure their LF accumulation. Nile red is a dye with a strong affinity to lipid tissues. Captured images were split into three sub-images based on the RGB palette. Only the red sub-image was analyzed to avoid the interference from auto-fluorescence (Supplementary Fig. 7 and Note 2). The analysis indicated that the LF accumulation of both the untreated N2 and CL2120 worms increased progressively from L3 to adult day 6 (Fig. 4a,b). Furthermore, the LF accumulation of exercise-treated N2 and CL2120 worms was significantly mitigated. The decreased LF served as a cue of boosted ROS removal in the worm’s body. Notably, near adult days 1 and 6 reported two surges of LF levels. We reason that the surges of LF levels may be subject to the maturity of the gonad, because the worms started to lay eggs at adult day 1 and ceased reproduction at adult day 6. Moreover, prior studies have shown that reproduction may regulate the proteostasis^26^,^27^,^28^,^29^. However, the comprehensive mechanism remains unclear.

A survivorship assessment for both N2 and CL2120 strains was conducted (Fig. 4c). All worms were censored during the process, and those expressing the bag of worm phenotype were excluded from the statistics. Compared with the untreated worms, the lifespans of exercise-treated N2 and CL2120 worms were 23 and 19 days, respectively. Thus, exercise-treated N2 and CL2120 worms can live 2 and 4 days longer than their control counterparts, respectively. The maximal lifespans of N2 and CL2120 worms also reached 30 and 26 days through the 4- and 6-day extensions, respectively. For the progeny assessments, eggs laid by the N2 and CL2120 worms were counted daily from adult days 1 to 9 (Fig. 4d). The average progeny numbers of the untreated N2 and CL2120 worms were 251 and 223, respectively. In contrast, the average progeny numbers of the exercise-treated N2 and CL2120 worms were 281 and 279, respectively. Hence, the exercise-treated worms can lay more eggs than the untreated worms. This perspective is confirmed by fewer instances of bag of worms in the exercise-treated CL2120 (Supplementary Fig. 8). According to the measurement, the ratio of bag of worm in the untreated worms was 34.2%, whereas, the ratio dropped to 26.4% for the exercise-treated worms at adult day 3. Moreover, the ratio of bag of worm in the untreated worms was 37.1%, whereas the ratio in the exercise-treated worms became 29.2% at adult day 4. The mechanism related to the increased number of progeny in this study remains not fully understood. However, a possible scenario is likely referred to the lowered oxidative stress by the electrotactic exercise. Prior literature^30^ has indicated a positive correlation between various stressors and reduced fertility. Oh *et al*.^31^ reported that progeny of *C. elegans* was increased when taking up a strong cellular antioxidant, N-acetyl-L-cysteine, to lower environmental stressors. Accordingly, scavenging excessive ROS may contribute to extending lifespan and upregulating fertility.

An additional experiment was also conducted to clarify which of the electrotaxis and the pure swimming contributed significantly to improvement. To this end, N2 worms were exposed to an electric field for 10 min while immobilized in a hydrogel (24% w/w Pluronic F127 solution) at every developmental stage from L4 to adult day 9 (Supplementary Fig. 9). Under the same treatment conditions (Fig. 1b), the treated and untreated worms exhibited no significant differences in terms of LF intensity. The experiment thus verifies that the major improvement can be attributed to the exercise itself and not the electrotaxis.

The antioxidant defense system can be enhanced by raising oxidative stress through regular exercise based on the model of oxidative stress and antioxidant (Supplementary Fig. 1). A periodic stimulation will then boost antioxidants to high levels. Previously, decreased LF accumulation implies low oxidative stress in exercise-treated worms. In addition, measuring the ROS level can directly support the hypothetical model. The red dye, 2,7-Dichlorofluorescin diacetate (DCF-DA), staining H_2_O_2_ was used herein to investigate the ROS level in the untreated and exercise-treated N2 and CL2120 worms (Fig. 4e,f). At least 50 worms were sonicated and stained with DCF-DA for each measurement to obtain sufficient signals on a microplate reader (Infinite 200 Pro, TECAN). Compared with the untreated N2 and CL2120 worms, the analysis showed that the ROS levels in the exercise-treated worms immediately soared after the exercise but plunged after 6 h at L4 and adult day 4. We reason that exercise increases oxygen consumption because of the increased mitochondria, thus resulting in the rise of free radical formation from these organelles. These surges further show that exercise initially brings a strong oxidative stress into the worms, followed by a rise of antioxidant enzymes, such as superoxide dismutase (SOD) and catalase (CAT).

Two mutant strains, GA480 (*sod-2 and sod-3* deficiency) and LB90 (*ctl-2* deficiency), were used to prove the abovementioned finding. Both strains were treated with exercise at 10 min each time from the L3 stage to adult day 6. Given that the GA480 and LB90 worms lacked the essential antioxidant enzymes, the exercise training caused extra oxidative burdens to them (Supplementary Fig. 10). Notably, the ROS damage on GA480 was more serious than that on LB90 because SOD dominated the upstream of the antioxidant pathway (Supplementary Fig. 1). Interestingly, the ROS level of LB90 showed a minimal decrease at the L4 stage. We reason that the short improvement is likely a sign of the upregulation of glutathione peroxidase (GPx). Nevertheless, the rebalance eventually failed at adult day 6 as the oxidative stress from the exercise remained. The simple experiment corroborated their roles in our exercise model. The boosted antioxidant enzymes then scavenge the excessive stress by suppressing the ROS level. This result is consistent with the previous LF assay. Collectively, extra antioxidants induced by constant exercise can maintain the ROS at a low level. However, exercise itself is a means by which to evoke oxidative stress, which can damage the worms before the antioxidants take effect. Conversely, inconstant, irregular, or over exercise is not only adverse to the improvement of degeneration, but also harmful to the worm’s body. Moreover, after comparing the ROS levels between the untreated N2 and CL2120 worms, the oxidative stress in CL2120 worms proved to be stronger than in N2 worms after the onset of Alzheimer’s disease. Without appropriate intervention, the escalated oxidative stress in the CL2120 worms as a result of accumulated Aβ can lead to paralysis in the early adult stage.

## Discussion

Physical exercise has long been recognized a simple measure to prevent degenerative diseases. However, owing to its difficult execution, the biological effect of exercise has never been fully studied on the model animal, *C. elegans*. To the best of our knowledge, the flow chamber coupling with a DC electric field provides a robust means of driving worms on demand for the first time. Consequently, the tiny animals can be used as a model to decipher the mystery behind such a mechanism. Taking advantage of the electrotaxis of *C. elegans*, we kept worms constantly swimming in the flow chamber for a prescribed duration by simply switching the electrical polarity alternately. Two major strains, N2 (wild-type strain) and CL2120 (Alzheimer model strain), were used to investigate their responses to the exercise treatment. The treatment was conducted for 10 min every day from L3 to adult day 6. The body wall sarcomeres of the worms were reinforced through a repeated damage-recovery process. The appearance of mitochondria in the body wall sarcomeres increased in response to the increased demand for muscular power during exercise. Finally, both the exercise-treated N2 and CL2120 worms showed significant improvements in muscle-related activities. For example, the kinetic power of the worms escalated, and the paralysis progress of CL2120 was also procrastinated.

Reduced oxidative stress benefited the lifespan and progeny of the worms. Muscle rejuvenation also delayed the cessation of reproduction and prevented the occurrence of bag of worm. Thus, all the improvements made herein extended the lifespan of the worms. After the exercise treatment, the N2 and CL2120 worms prolonged their average lifespans from 21 days and 15 days to 23 days and 19 days, respectively. This result indicates that the exercise treatment can enable CL2120 worms to live up to three weeks, nearly the same as the normal N2 worms, after its onset. When LF and ROS are considered, this tendency supports the hypothetical model of oxidative stress and antioxidant. LF is widely known as a cytotoxic byproduct of ROS. Excessive LF triggers cell apoptosis and leads to body function degeneration. A high ROS level usually goes with dense LF granules. The LF accumulation in the exercise-treated N2 and CL2120 worms declined significantly. Although the overall degeneration trend is irreversible, the degenerative progress can be certainly mitigated. Thus, the exercise treatment proved effective to both healthy (N2) and unhealthy (CL2120) subjects because their senescence processes were regulated by the exercise treatment. Similarly, overall ROS levels dropped in both the exercise-treated N2 and CL2120 worms. However, the ROS levels surged right after exercise and then plunged after the antioxidant enzymes (SOD and CAT) were induced. ROS directly supports the variation of oxidative stress in *C. elegans*. As a result, suppressing oxidative stress can benefit the improvement of degeneration.

In this study, we successfully proved the positive effects of exercise in improving age-related degeneration and delaying the progress of Alzheimer’s disease by using the nematode *C. elegans* as a model animal. A self-developed flow chamber coupled with a DC electric field was used to conduct the experiment. The findings provided solid support for the hypothetical model of oxidative stress and antioxidant. In addition, the flow chamber can be used to study the relationship between exercise and biological signaling pathways as well as the role of the nervous system in the regulation of degeneration in the future.

## Methods

### *C. elegans* culture

Bristol N2 was used as the wild-type strain. All other *C. elegans* strains were requested from the Caenorhabditis Genetics Center (CGC), supported by the National Institute of Health, Office of Research Infrastructure Programs (P40 OD010440). The worms were maintained at 20 °C in an incubator and bred on agar dishes seeded with *Escherichia coli* strain OP50 as a food source according to standard protocols^32^. The transgenic worms CL2120 [*unc-54p::beta 1–42*(pCL12) + *mtl-2p::*GFP (pCL26)] express the human Aβ1–42 peptide under the control of the *unc-54* (myosin heavy chain) promoter that drives expression to the body wall muscle cells. These animals constitutively formed intracellular amyloid deposits in their muscle cells. The strain has the *mtl-2*p::GFP as a marker gene; hence, it expresses GFP in the intestinal cells. Aβ toxicity is enhanced at higher temperatures, thus, the worms were grown and maintained on fresh 60-mm NGM agar seeded with *E. coli* OP50 at 15 °C.

Others transgenic strains were used for stress assay, mitochondria density, and sarcomere evolution. TJ356 [*daf-16p::daf-16a/b::GFP*+ *rol-6*] expresses GFP in DAF-16. Nuclear localization responded to environmental stimuli, such as starvation, heat shock, and oxidative stress^33^. We used this strain to inspect the stress in response to hot bath and electrotaxis. The RW1596 [*myo-3p::GFP::myo-3* + *rol-6*(*su1006*)] is a transgenic strain engineered with GFP in sarcomeres, whereas the SJ4103 [*myo-3p*::GFP(mit)] is a transgenic strain that expresses GFP in the mitochondria of body wall muscle cells. All of the transgenic worms were maintained at 20 °C. RW1596 was used to measure the sarcomere changes, and SJ4143 was used to monitor the mitochondria generation regulated by exercise. LB90 [*ctl-2*(*ua90*) *II*; *him-8*(*e1489*) *IV*] was used to assess the role of the CAT enzyme in the oxidative stress. This mutant strain is a *cat* deficient model that exhibits a shortened lifespan. GA480 [*sod-2*(*gk257*) *I; sod-3*(*tm760*) *X*] was used to assess the role of the SOD enzyme in the oxidative stress. This mutant strain is characterized by slow growth, reduced brood size, and hypersensitivity to oxidative stress.

### Statistical analysis

Data from independent measurements are presented in the figures as mean ± SEM. One-way student *t* test was performed to determine whether a significant difference between the two groups existed. Here, *, **, and *** were used to indicate p values less than 0.05, 0.01, and 0.001, respectively; otherwise, the comparison was labeled as no significant difference (N.S.).

### Lifespan assay

For the lifespan assay, the tested worms were moved to new 6-mm agar dishes, which were seeded with *E. coli* OP50, and were marked as day 0 when growing to the L4 stage. Similarly, the worms were transferred to new agar dishes daily during the first 4–5 days to avoid mixing between generations. The worms that showed no response to gentle prodding with a platinum wire and no pharyngeal pumping were considered dead. All worms were censored during the process and those expressing the bag of worm phenotype were excluded from the statistics. Survivorship was analyzed using GraphPad Prism 5.0 (GraphPad Software, La Jolla Inc.). The Mantel-Cox log rank test was used to assess the statistical significance between different curves of survival rates. The difference was considered significant only when p value was less than 0.01.

### Progeny size assay

Adult worms were transferred daily to fresh 60-mm agar dishes seeded with *E. coli* OP50. Next, the number of eggs left on the old agar dishes was immediately counted. To prevent human errors, each plate was counted thrice for the total number of progeny. The mean progeny number each day was eventually derived from dividing the total number of progeny by the total number of worms.

### Nile red staining

Nile red, a dark purplish dye (N-3013, Sigma) that appealed to lipids, was used as an ideal lipophilic dye to locate LF accumulation in the worm’s body. For a stock solution, 1 mg of Nile red power was dissolved in a 1000 μL aqueous medium of Dimethyl sulfoxide (DMSO). The stock was diluted with an M9 buffer at a ratio of 1:1000, which subsequently resulted in the diluted stain solution. Selected worms were added into the Eppendorf. After 60 min, the stained worms were washed thrice with an M9 buffer^34^,^35^,^36^.

For the quantitative analysis of LF accumulation, the stained worms were placed on a glass slide coated with a thin film of 2% agarose gel. To fix the worms, a drop of M9 buffer containing 1% sodium azide was pipetted over the worms, which were then covered with a coverslip. The glass microchip was then placed under a fluorescent microscope equipped with a green filter cube (excitation at 450 nm to 490 nm, with a 505-nm dichroic mirror and a 520 nm barrier filter) and a 10× objective for measurements. Fluorescent images were captured using a digital camera (DP72, Olympus). Red image slices were extracted from the images based on their color histogram (RGB) using ImageJ. The final LF level was defined as an average pixel intensity of the region of interest (Supplementary Fig. 7 and Note 2).

### ROS assay

Intracellular H_2_O_2_ was stained by a 2,7-Dichlorofluorescin diacetate (DCF-DA). For the stock solution, 0.5 mg DCF-DA powder was dissolved in a 1000 μL aqueous medium of DMSO. The stock solution was then diluted with a buffer composed of phosphate buffered saline (1× PBS) and 1% tween-20 (ex: 95 μl PBS and 5 μl tween-20) in an eppendorf tube. The worms (n = 50) were collected in an Eppendorf tube and then sonicated until their cells were totally ruptured for measurement. After dispensing the solution into a 96-well plate, the stained liquid samples were read at room temperature (about 25 °C) every 10 min for 2 h by using a microplate reader (Excitation 485 nm/Emission 535 nm, Molecular Devices)^37^. The data measured at 30 min were used for subsequent comparisons and analyses^38^.

### Derivation of kinetic power from swimming *C. elegans*

A colloidal suspension was prepared by mixing 40-μL tracer particles (d_p_ = 3 μm, 540/610, Fisher Scientific) in a 1-mL NGM buffer. A 0.5 μL droplet containing a worm was sandwiched between two glass slides separated with a 110 μm spacer. Mineral oil (Acros Organics) covered the droplet to prevent evaporation. Given that the waist diameter of an adult worm is around 80–90 μm, the gap is merely sufficient for a worm to swim in plane. As a result, out-of-plane movement is negligible. According to the principle of energy conservation, the movement of the worm is the only source of perturbation in the liquid in such an isolated droplet. Therefore, the calculated kinetic power of the liquid droplet is equivalent to the power exerted from the worm. In this way, an acceleration field can be derived from the velocity fields. The kinetic power of the worm can then be estimated from the acceleration field^22^.

A 10× objective and a set of filters corresponding to the fluorescent particles were used in this study. Under the fluorescent mode, a dim background light from the top condenser was required to identify the worm profile. A high-speed camera (GX-3, NAC) was used to acquire consecutive particle images. A frame rate of 50 Hz was typically sufficient to capture the N2 locomotion. Total acquisition duration was set at 2 s to allow the inclusion of at least 3 complete swimming cycles. Velocity fields were derived from the captured images using the cross-correlation algorithm. Detailed information on μPIV are provided in the relevant literature^23^,^24^,^39^.

### Electrotaxis of immobilized *C. elegans*

An open microchannel measuring 25, 5, and 2 mm in length, width, and depth, respectively, was used for this experiment. The microchannel consisted of polymethyl methacrylate (PMMA) and was fabricated by a CNC mill (EGX-400, Roland). The bottom of the microchannel was sealed with a cover glass using a UV adhesive (Prime & Bond NT, Dentsply), followed by a UV light cure for 24 h. The channel was then filled with a 24% w/w Pluronic F127 solution. A thermoelectric (TE) cooler was placed under a microchannel to maintain a low temperature (<15 °C) when the hydrogel solution was loaded. The synchronized worms were subsequently washed from the culture agar plate and poured on to the hydrogel solution. Once all worms were immersed into the hydrogel solution, the TE cooler was removed to induce worm immobilization by solidifying the hydrogel. Two electrode wires were inserted at the two ends of a straight microchannel. The effective electric intensity of electrotaxis was selected according to the developmental stage of the worm (Fig. 1b); it was applied to the hydrogel solution for 10 min at each time afterward. After the treatment, a TE cooler was used to liquidize the hydrogel. By swiftly transferring the worms and the hydrogel solution to a new agar plate with a lawn of *E. coli* using a pipette, the worms crawled out of the hydrogel and were measured for their LF intensity. This procedure was repeatedly imposed on worms from L4 to adult day 9.

## Additional Information

**How to cite this article**: Chuang, H.-S. *et al*. Exercise in an electrotactic flow chamber ameliorates age-related degeneration in *Caenorhabditis elegans*. *Sci. Rep.* **6**, 28064; doi: 10.1038/srep28064 (2016).

## Supplementary Material

Supplementary Video S1

Supplementary Information
