# Supplementary material for: Exercise in an electrotactic flow chamber ameliorates age-related degeneration in Caenorhabditis elegans
Source: Sci Rep. 2016 Jun 16;6:28064. doi: 10.1038/srep28064 (PMC4910109; doi:10.1038/srep28064)
Supplement: Supplementary Information [file srep28064-s2.pdf]

Exercise in an electrotactic flow chamber ameliorates age-related degeneration in *Caenorhabditis elegans*

Han-Sheng Chuang[1,2,*], Wan-Jung Kuo[1], Chia-Lin Lee[1], I-Hua Chu[3], and Chang-Shi Chen[4]

[1] Department of Biomedical Engineering, National Cheng Kung University, TAIWAN

[2] Medical Device Innovation Center, National Cheng Kung University, TAIWAN

[3] Department of Sports Medicine, Kaohsiung Medical University, TAIWAN

[4] Department of Biochemical and Molecular Biology, National Cheng Kung University, TAIWAN

[*] oswaldchuang@mail.ncku.edu.tw.

**Supplementary Figure 1**	Concept of antioxidant enzymes elevated by exercise.
**Supplementary Figure 2**	Configurations of the flow chamber and the experimental setup.
**Supplementary Figure 3**	Electrotaxis of free swimming *C. elegans*.
**Supplementary Figure 4**	Score definitions for the body wall sarcomeres in the transgenic strain, RW1596.
**Supplementary Figure 5**	Mean diameter of mitochondrion in the transgenic strain, SJ4103.
**Supplementary Figure 6**	Measurement of kinetic power of *C. elegans*.
**Supplementary Figure 7**	Quantification of the LF accumulation in *C. elegans*
**Supplementary Figure 8**	Bag of worm for the untreated CL2120 worms.
**Supplementary Figure 9**	Electrotaxis of immobilized *C. elegans*
**Supplementary Figure 10**	Oxidative stress of the antioxidant enzyme deficient mutants
**Supplementary Note 1**	Model of oxidative stress and antioxidants
**Supplementary Note 2**	Image assessment of LF accumulation
**Supplementary Video S1**	Swimming in the worm treadmill

Note: Supplementary Video is available on the journal website.

Supplementary Figure 1

Concept of antioxidant enzymes elevated by exercise.

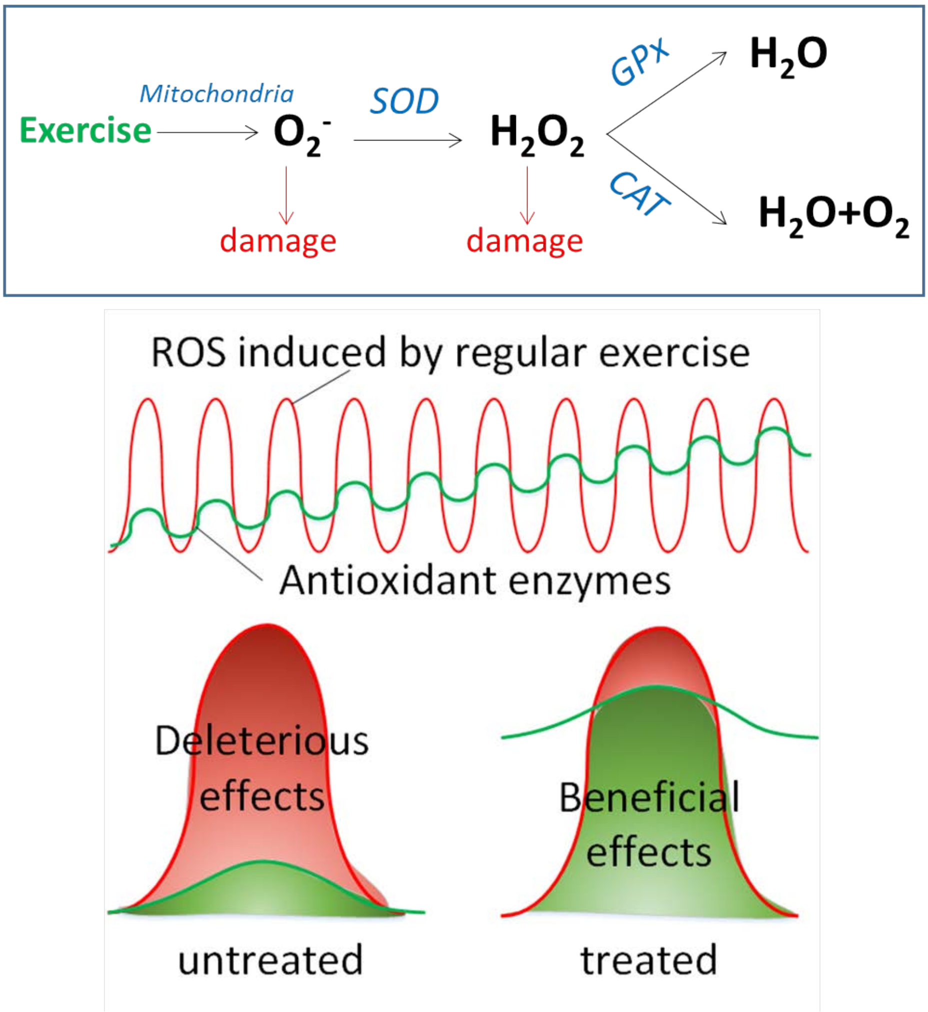

The formula in the box shows the redox of oxidative stress and antioxidant enzymes (superoxide dismutase (SOD), catalase (CAT), and glutathione peroxidase (GPx)) in cells. According to the formula, the level of antioxidant enzymes can be progressively elevated by inducing oxidative stress through regular exercise. For the untreated worms, the deleterious effects (upper red region) surpass the beneficial effects (lower green region) in cells, resulting in degenerative diseases. For the exercise-treated worms, however, the beneficial effects are higher than the deleterious effects, resulting in procrastinating degeneration.

Supplementary Figure 2

Configurations of the flow chamber and the experimental setup.

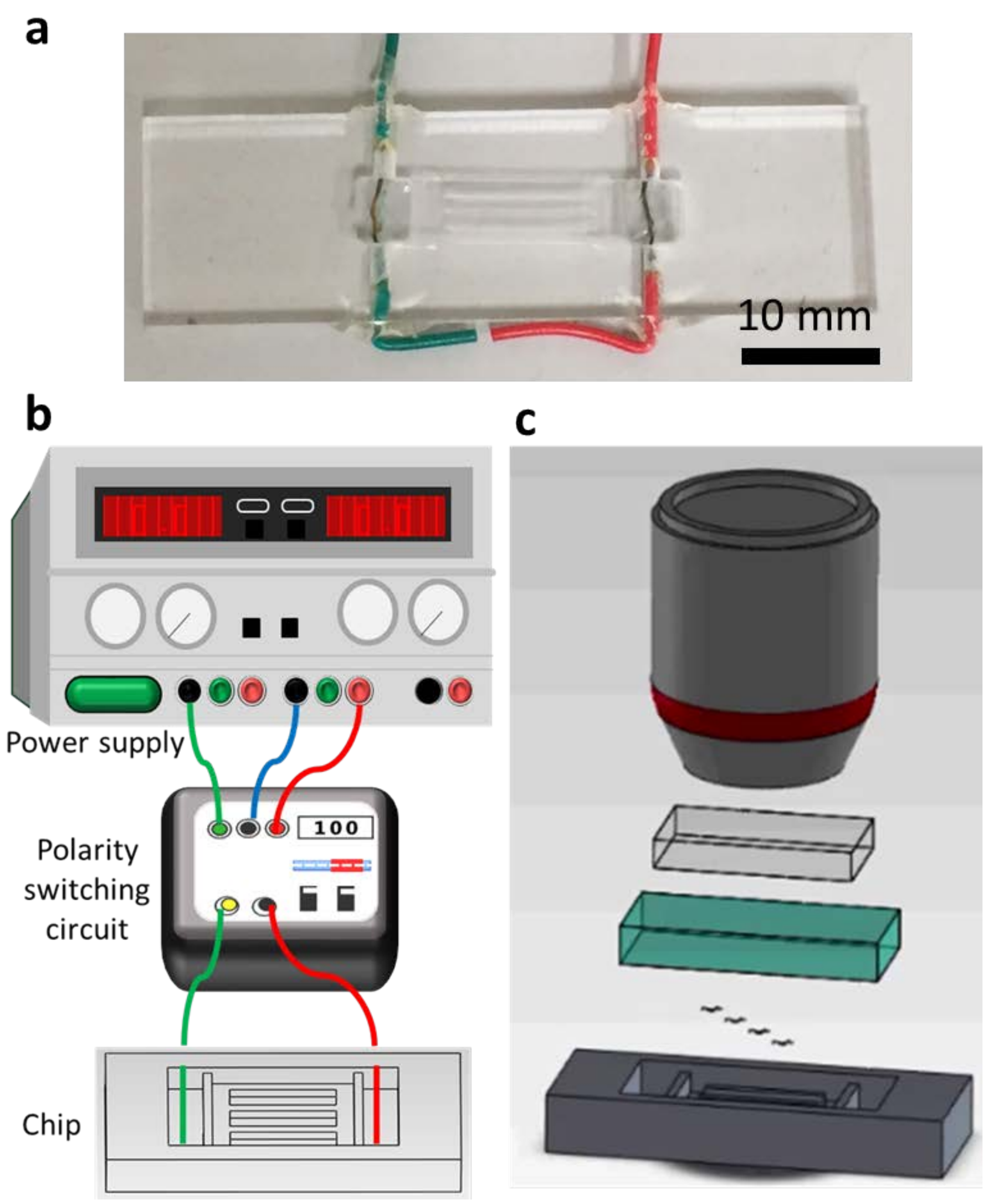

(**a**) Flow chamber is used to drive *C. elegans* to swim in the microchannels with electrotaxis. The distance between the two parallel electrical wires is 21 mm. The electrical wires are covered with agarose gel to prevent air bubbles. (**b**) Schematic of the experimental setup. The DC power supply provides a positive voltage, a negative voltage, and a ground to the polarity switching circuit. The circuit then outputs a periodically switching electrical polarity to the flow chamber to confine the swimming worms within the microchannels. (d) Assembly drawing of the flow chamber with worms under an objective lens. The status of the worms under a treatment is monitored in real time by a microscope.

Supplementary Figure 3

Electrotaxis of free swimming *C. elegans*.

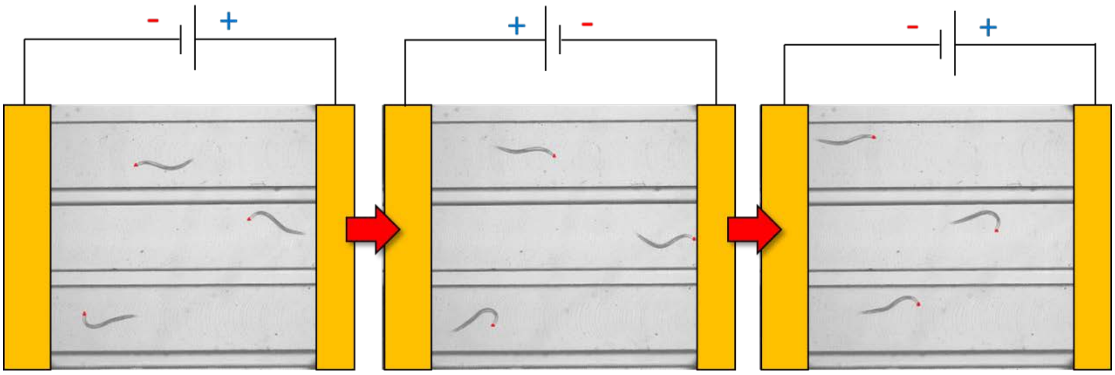

By dynamically switching the electrical polarity, the worms are driven toward the cathode. The red dots indicate the heads of the worms. The consecutive images show that the three worms are confined within the region of interest in a time duration by simply switching the electrical polarity.

Supplementary Figure 4

Score definitions for the body wall sarcomeres in the transgenic strain, RW1596.

a

Sarcomere image	Features	Score
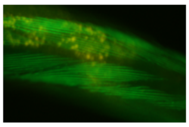	Tight, parallel, and smooth	0
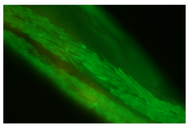	Slightly less dense, irregular orientation or not smooth	1
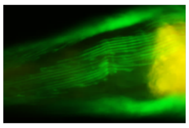	Banding or uneven in shape	2
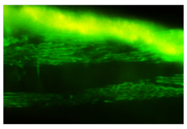	Wave-like shapes, such as '-u-' or minor rupture	3
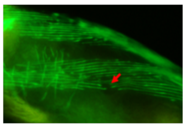	Fibers rupture and randomly distributed	4

b

	Score for sarcomere integrity				
Stage	N=1	N=2	N=3	N=4	N=5
Adult Day 2_control	0	0	0	0	0
Adult Day 2_exercise	0	0	0	0	0
Adult Day 3_control	1	0	0	0	0
Adult Day 3_exercise	2	1	1	1	1
Adult Day 4_control	2	1	1	0	0
Adult Day 4_exercise	2	1	1	1	1
Adult Day 5_control	2	2	1	1	0
Adult Day 5_exercise	2	2	2	1	0
Adult Day 6_control	3	1	1	1	1
Adult Day 6_exercise	2	2	1	1	1
Adult Day 7_control	2	2	1	1	1
Adult Day 7_exercise	2	1	1	1	0
Adult Day 8_control	2	2	1	1	1
Adult Day 8_exercise	2	1	1	1	1
Adult Day 9_control	3	2	1	1	1
Adult Day 9_exercise	2	2	1	1	1
Adult Day 10_control	3	3	2	2	1
Adult Day 10_exercise	3	2	1	1	1
Adult Day 11_control	2	2	2	2	1
Adult Day 11_exercise	2	2	2	2	1
Adult Day 12_control	3	2	2	2	1
Adult Day 12_exercise	2	2	2	2	1
Adult Day 13_control	3	3	2	2	2
Adult Day 13_exercise	3	2	2	2	1
Adult Day 14_control	3	3	2	2	2
Adult Day 14_exercise	3	2	2	2	1
Adult Day 15_control	4	4	3	2	2
Adult Day 15_exercise	3	2	2	2	2

c

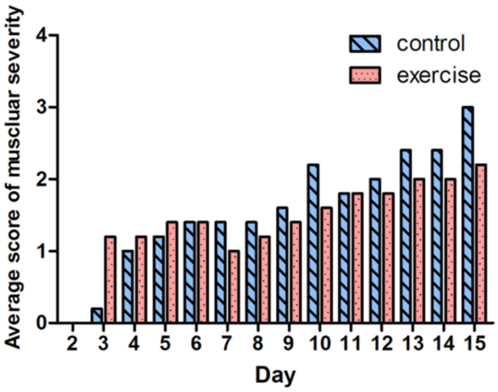

d

Total Point	Control	Exercise
Adult day 2	0	0
Adult day 3	1	6
Adult day 4	5	6
Adult day 5	6	7
Adult day 6	7	7
Adult day 7	7	5
Adult day 8	7	6
Adult day 9	8	7
Adult day 10	11	8
Adult day 11	9	9
Adult day 12	10	9
Adult day 13	12	10
Adult day 14	12	10
Adult day 15	15	11

Score of sarcomere integrity (n=5). The worms were treated with exercise from L3 to adult day 6. The sarcomere images were captured and assessed at 6 h after every exercise treatment from adult day 2 to adult day 15. (**a**) Score 0 represents organized and smooth muscle fibers; whereas score 4 represents ruptured and disorganized muscle fibers. The high scores usually happen to old adult worms or worms after a vigorous exercise treatment. (**b**) The table reveals the detailed records of the RW1596 worms with and without the exercise treatment from adult day 2 to adult day 15. (**c**) Degree of sarcomere integrity. The exercise-treated worms indeed show a lower score than the untreated worms, implying a healthier muscle status. (d) The total scores of the exercise treated and untreated worms every day.

Supplementary Figure 5

Mean diameter of mitochondria in the transgenic strain, SJ4103.

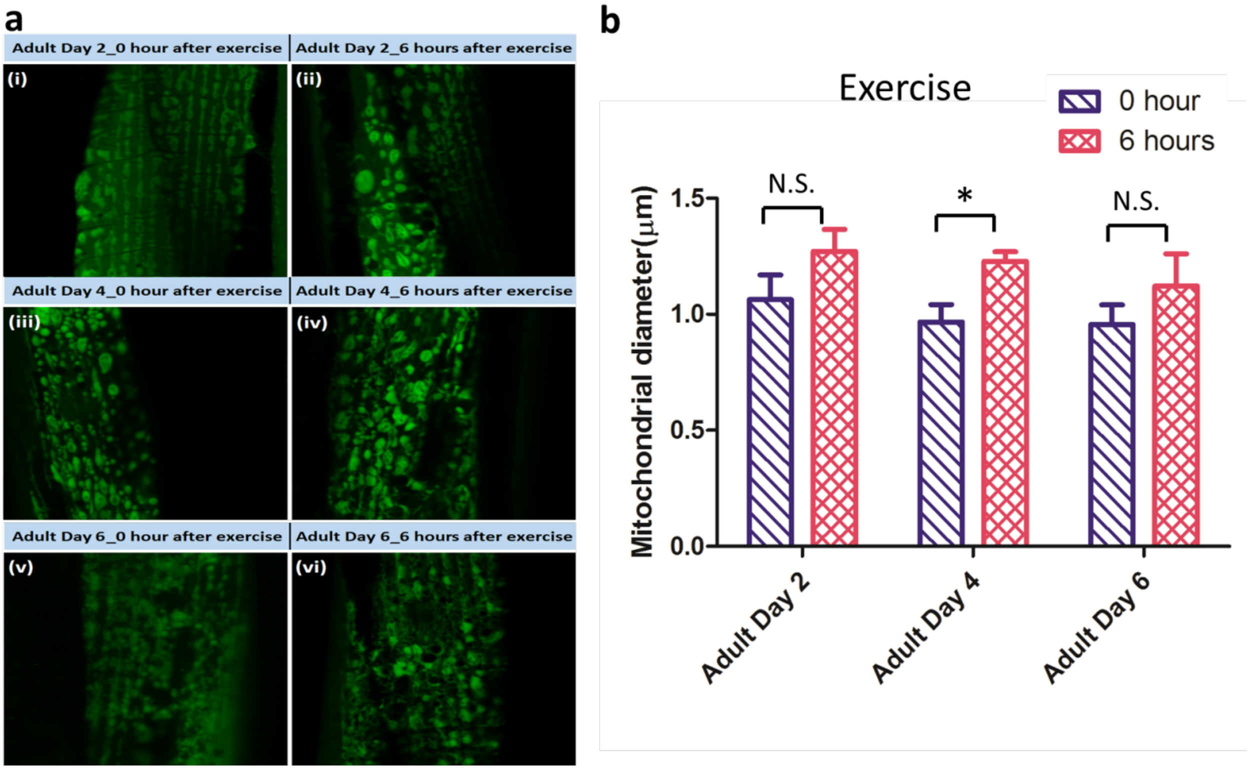

(**a**) Confocal images of mitochondria in the body wall muscle cells at adult day 2, day 4, and day 6 measured at 0 and 6 h after each exercise. The mitochondria are tagged with GFP and appear in granular shapes. (**b**) Plot of mean diameter of mitochondrion (n=40). Except adult day 6, larger and denser mitochondria obviously appear at 6 h after the exercise. At the later developmental stage (adult day 6), the mitochondrial fusion becomes less efficient to make up for the impairment due to age-related degeneration.

Supplementary Figure 6

Measurement of kinetic power of *C. elegans*.

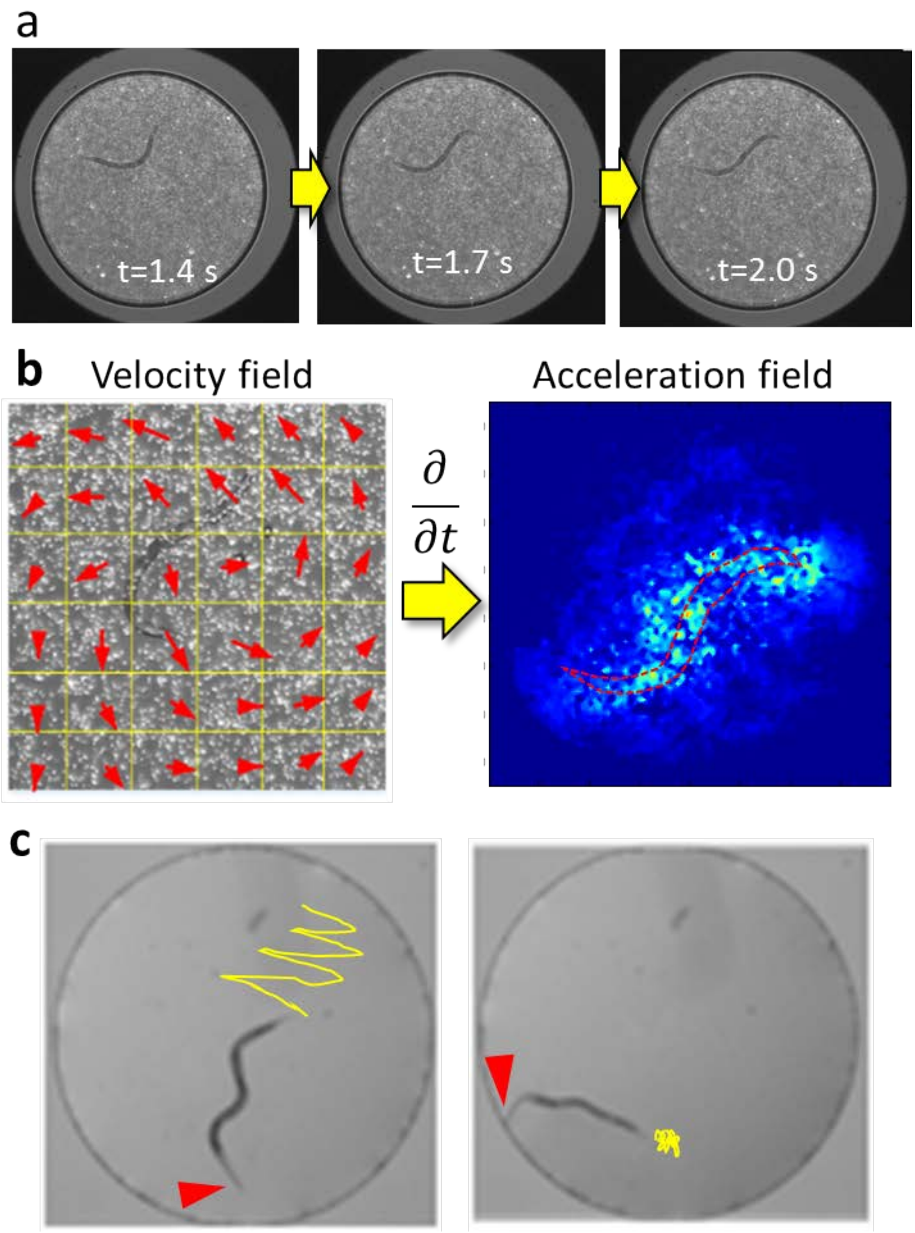

(**a**) Consecutive images of a N2 worm swimming in an isolated droplet. The droplet is suspended with 3 μm fluorescent polystyrene particles. The droplet is sandwiched between two glass slides separated with a 110 μm spacer. (**b**) A velocity field is obtained by analyzing the particle displacements using the spatial cross correlation algorithm. Subsequently, two velocity fields can generate an acceleration field. The final acceleration field can be used for the calculation of kinetic power. (**c**) Paralysis of a worm is distinguished by the trajectory of the worm's tail (yellow line). The red arrow indicates the head of a worm. When the worm is normal, the yellow line will appear to be zig-zag (left); otherwise, the yellow line will turn out to be a tangle (right).

Supplementary Figure 7

Quantification of the LF accumulation in *C. elegans*.

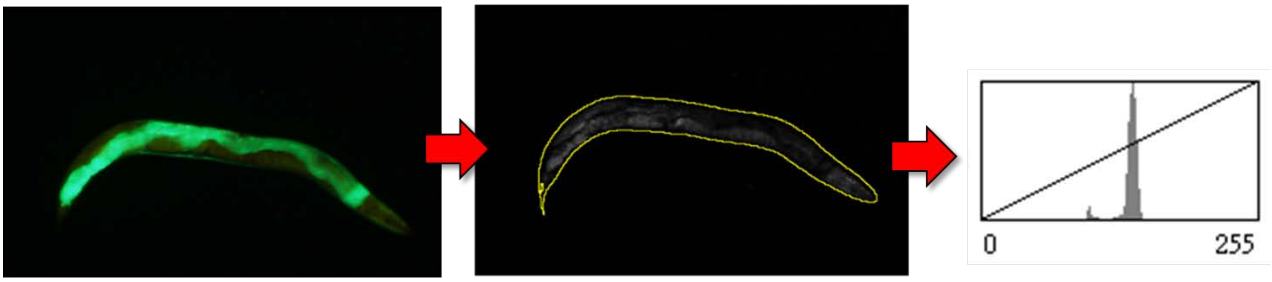

The fluorescent image is captured using a digital camera (DP72, Olympus) and an inverted microscope (IX71, Olympus) equipped with a green filter cube (excitation at 450-490 nm, a 505 nm dichroic mirror, and a 520 nm barrier filter) and a 10x objective. The captured color image is split into three sub-images based on the RGB palette. To avoid interference from the auto-fluorescence, only the red sub-image is analyzed. The final quantification is obtained by dividing the sum of the total gray levels by the selected region (the solid yellow line).

Supplementary Figure 8

Bag of worm for the untreated CL2120 worms.

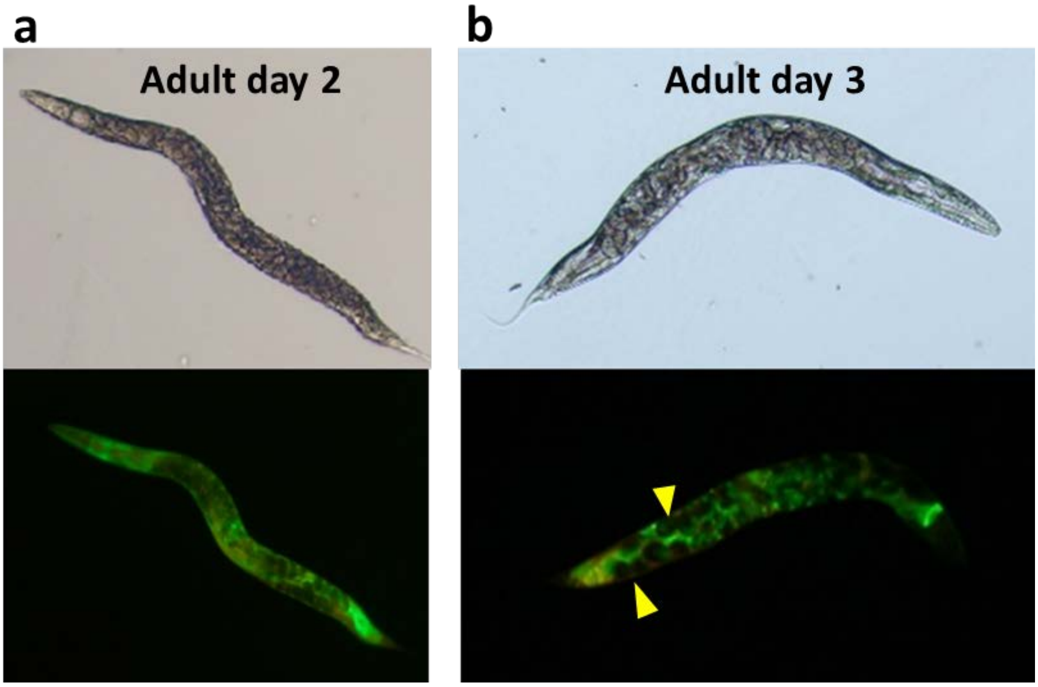

(**a**) Appearances of the untreated worm at adult day 2 in the white-light mode (upper) and the fluorescent mode (lower). (**b**) Appearances of the same worm at adult day 3 in the white-light mode (upper) and the fluorescent mode (lower). The yellow arrows indicate some of the eggs trapped in the worm's body. Compared to the worm at adult day 2, more eggs are found congested in the body, which causes the worm to dilate in size.

Supplementary Figure 9

Electrotaxis of immobilized *C. elegans*

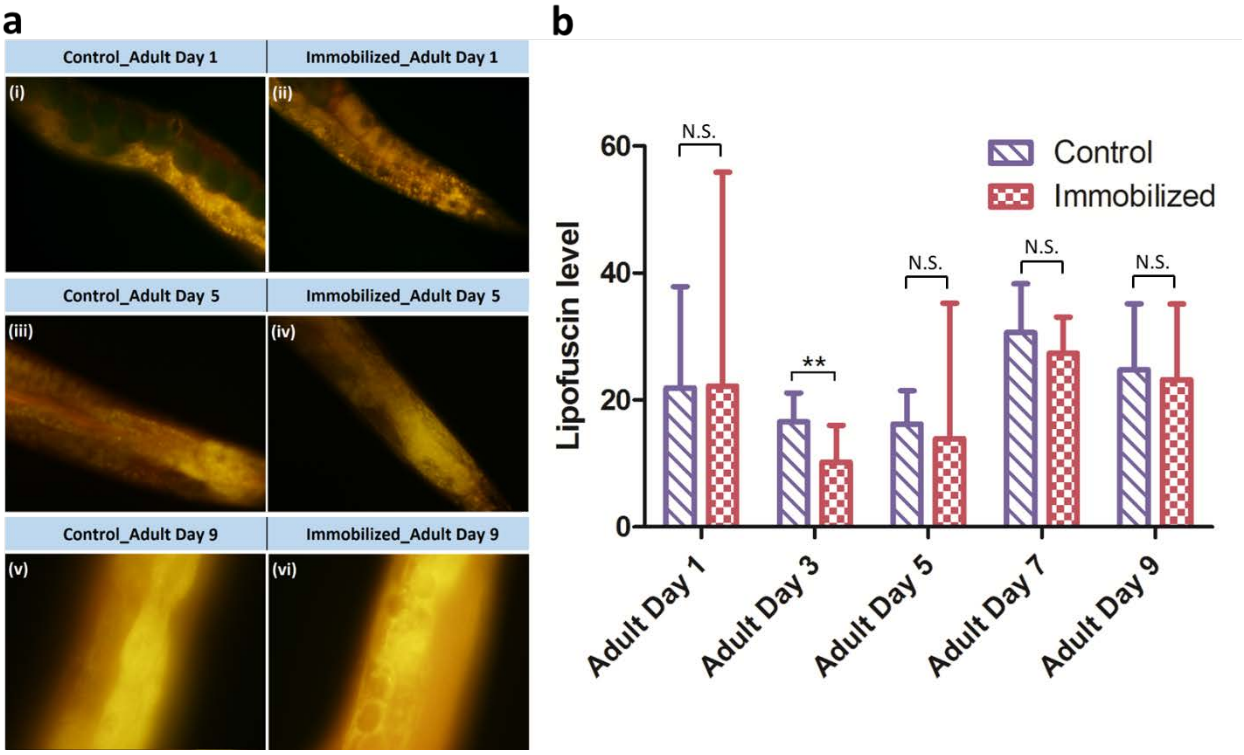

(**a**) Fluorescent images of N2 worms stained with the Nile red dye (n=15). The LF levels at all developmental stages between the treated (immobilized) and untreated (control) worms are very close. Notably, the treated worms were exposed to an electric field when being immobilized in a hydrogel at the same time. (**b**) Statistical plot of the LF intensity of the treated and untreated N2 worms. Except adult day 3, all other developmental stages show no significant differences. The result implies that electrotaxis hererin may play an insignificant role in altering the antioxidants in the model of oxidative stress.

Supplementary Figure 10

Oxidative stress of the antioxidant enzyme deficient mutants

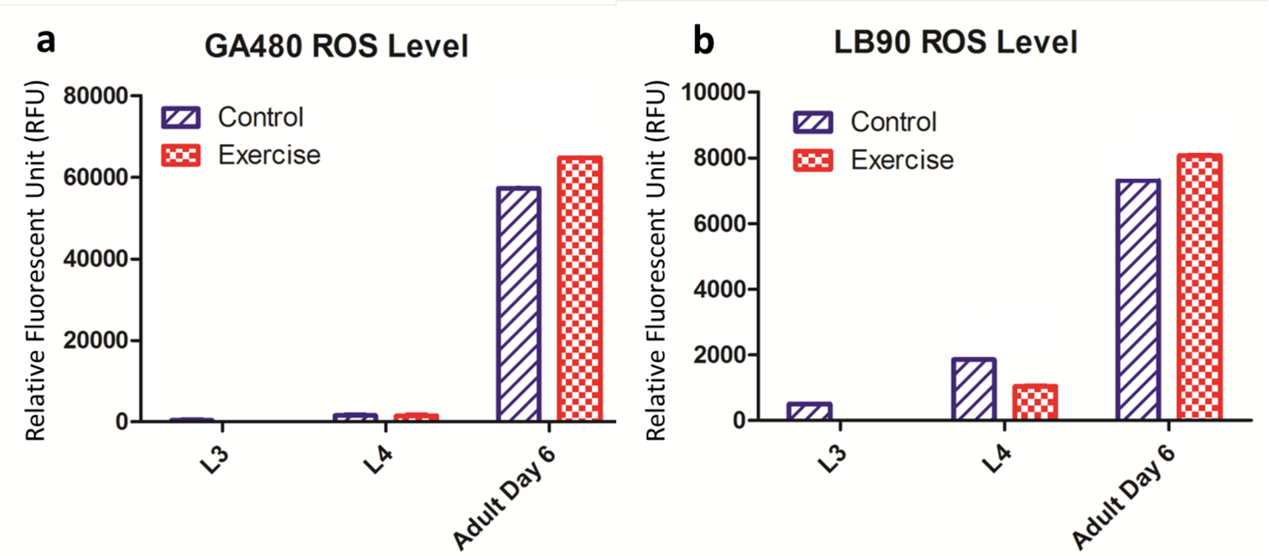

Plots of ROS levels of the GA480 (n=50) and LB90 (n=50) mutant worms. For the both strains, ROS surges at later developmental stages. Exercise-treated worms show higher oxidative stress than their counterparts at adult day 6. The SOD deficient strain, GA480, bears higher oxidative stress than LB90. The trend corroborates that SOD and CAT enzymes play important roles in the ROS scavenging process. In each case, 50 worms were sonicated and stained with DCF-DA to reveal their overall ROS levels on a microplate reader.

Supplementary Note 1

Model of oxidative stress and antioxidants

The effect of exercise on the improvement of age-related degeneration herein relies on the hypothetical model of oxidative stress and antioxidants. According to this model, oxidative stress is a measure to awake the protection mechanism hiding in cells. Oxidative stress, originated from free radicals, such as ROS, is well known adverse to the cell activities. Under the attack of free radicals, cells will accordingly be forced to release higher doses of antioxidant enzymes to scavenge the excessive ROS. By appropriately stimulating cells with high oxidative stress, the concentrations of antioxidant enzymes will be maintained at higher levels due to adaptation. Therefore, ROS will be suppressed at a low level in the trained cells, preventing them from the damage of free radicals. Since ROS impedes cell growth and causes cell apoptosis, a low ROS environment benefits the cell activities and lifespan. Ironically, however, living organisms cannot live completely without oxidative stress either. Minor stress seems of vital help to keep cells functioning normally. In this case, certain heat shock proteins are induced to aid living organisms to survive in an adverse condition.

Despite numerous ways to raise oxidative stress, physical exercise remains the natural and simplest method to achieve this purpose. Exercise increases consumption of oxygen in response to the demand for energy. The high energy output is originated from the increase of mitochondria. Since H_2O_2 is produced from the dismutation of superoxide ($O_2^{\bullet -}$) generated within mitochondria, the oxidative stress is accordingly increased. To maintain homeostasis, cells will release antioxidant enzymes, such as superoxide dismutase (SOD), catalase (CAT), and glutathione peroxidase (GPx), to lower the ROS level. Long-lasting antioxidant enzymes can be induced by repeatedly stimulating cells with oxidative stress. The antioxidant enzymes can be maintained at a high level when cells adapt to the stress. In our hypothesis, we expect the same oxidative stress-antioxidant mechanism can be incurred in *C. elegans* by the regular exercise treatment (*i.e.*, the electrotaxis-driven swimming).

Supplementary Note 2

Image assessment of LF accumulation

To locate the LF distribution in *C. elegans*, worms were stained with Nile red solution for an hour firstly and then fixed on a glass slide with sodium azide. When the LF granules are stained with Nile red, they will exhibit in orange-yellow color, differing from the autofluorescent background. Consequently, more LF accumulation will result in larger and brighter stained regions shown in the worm's body. A 10x objective was used to ensure the entire worm to be included in an image. Moreover, a green filter cube (excitation at 450-490nm, a 505nm dichroic mirror, and a 520nm barrier filter) was used to enhance the fluorescent image. For quantification analysis, the image was split into three sub-images according to the RGB palette using the freeware ImageJ. At last, only the red sub-image was converted to a gray-level image to facilitate the subsequent analysis. At each developmental stage, 8 worms of CL2120 and 12 worms of N2 were sacrificed for the assessment of LF accumulation.

Each worm in the image was outlined and then summed up their intensity in the region of interest (**Supplementary Fig. 7**). An index showing the degree of LF accumulation was obtained by dividing the total intensity by the total area of the selected worm. To avoid the bias due to the background intensity of each image, the average background intensity was subtracted from the final index in an image.

Supplementary Video S1

Swimming in the worm treadmill

Three adult worms oriented by electrotaxis are separately swimming in the worm treadmill. A DC electric field is applied to the flow chamber with the negative pole on the right end of the microchannels and the positive pole on the left end of the microchannels at the beginning. Next, the polarity of the electric field is switched twice, causing the worms to reorient themselves with the negative pole. The video showcases that the worms can be driven to exercise within a desired timeframe on demand.
